# Engineering human ACE2 to optimize binding to the spike protein of SARS coronavirus 2

**DOI:** 10.1126/science.abc0870

**Published:** 2020-08-04

**Authors:** Kui K. Chan, Danielle Dorosky, Preeti Sharma, Shawn A. Abbasi, John M. Dye, David M. Kranz, Andrew S. Herbert, Erik Procko

**Affiliations:** 1 Orthogonal Biologics, Champaign, IL 61821, USA.; 2 U.S. Army Medical Research Institute of Infectious Diseases, Frederick, MD 21702, USA.; 3 Department of Biochemistry and Cancer Center at Illinois, University of Illinois, Urbana, IL 61801, USA.; 4 The Geneva Foundation, Tacoma, WA 98402, USA.

## Abstract

The spike (S) protein of severe acute respiratory syndrome coronavirus 2 (SARS-CoV-2) binds angiotensin-converting enzyme 2 (ACE2) on host cells to initiate entry, and soluble ACE2 is a therapeutic candidate that neutralizes infection by acting as a decoy. By using deep mutagenesis, mutations in ACE2 that increase S binding are found across the interaction surface, in the asparagine 90–glycosylation motif and at buried sites. The mutational landscape provides a blueprint for understanding the specificity of the interaction between ACE2 and S and for engineering high-affinity decoy receptors. Combining mutations gives ACE2 variants with affinities that rival those of monoclonal antibodies. A stable dimeric variant shows potent SARS-CoV-2 and -1 neutralization in vitro. The engineered receptor is catalytically active, and its close similarity with the native receptor may limit the potential for viral escape.

## A decoy receptor for SARS-CoV-2

For severe acute respiratory syndrome coronavirus 2 (SARS-CoV-2) to enter human cells, the spike protein on the surface of the virus must bind to the host receptor protein, angiotensin-converting enzyme 2 (ACE2). A soluble version of the receptor is being explored as a therapeutic. Chan *et al.* used deep mutagenesis to identify ACE2 mutants that bind more tightly to the spike protein and combined mutations to further increase binding affinity (see the Perspective by DeKosky). A promising variant was engineered to be a stable dimer that has a binding affinity for the spike protein; it is comparable with neutralizing antibodies and neutralized both SARS-CoV-2 and SARS-CoV-1 in a cell-based assay. In addition, the similarity to the natural receptor may limit the possibility for viral escape.

*Science*, this issue p. 1261; see also p. 1167

## Teaser

A variant of ACE2 based on deep mutagenesis far outcompetes the natural receptor in binding the SARS-CoV-2 spike protein.

In late 2019, a novel zoonotic betacoronavirus closely related to bat coronaviruses crossed into humans in the Chinese city of Wuhan (*1*, *2*). The virus, called severe acute respiratory syndrome coronavirus 2 (SARS-CoV-2) because of its similarities with the SARS coronavirus first discovered in 2003 (*3*, *4*), causes coronavirus disease 2019 (COVID-19) (*5*), which is producing devastation across the globe.

The spike (S) glycoprotein of SARS-CoV-2 binds angiotensin-converting enzyme 2 (ACE2) on host cells (*2*, *6*–*11*). S is a trimeric class I viral fusion protein that is proteolytically processed into S1 and S2 subunits that remain noncovalently associated in a prefusion state (*6*, *9*, *12*). Upon engagement of ACE2 by a receptor binding domain (RBD) in S1 (*13*), conformational rearrangements occur that cause S1 shedding, cleavage of S2 by host proteases, and exposure of a fusion peptide adjacent to the S2' proteolysis site (*12*, *14*–*16*). Folding of S to a postfusion conformation is coupled to host cell–virus membrane fusion and cytosolic release of viral RNA. Atomic contacts with the RBD are restricted to the extracellular protease domain of ACE2 (*17*, *18*). Soluble ACE2 (sACE2) in which the transmembrane domain has been removed is sufficient for binding S and neutralizing infection (*10*, *19*–*21*). A broad collection of highly potent neutralizing antibodies have been isolated (*22*–*28*), yet the virus spike shows rapid accumulation of escape mutations when under selection (*29*). By comparison, the virus may have limited potential to escape sACE2-mediated neutralization without simultaneously decreasing affinity for native ACE2 receptors, an outcome that is likely to attenuate virulence. Furthermore, sACE2 could potentially treat COVID-19 symptoms by proteolytic conversion of angiotensin peptides that regulate blood pressure and volume (*30*, *31*). Recombinant sACE2 is safe in healthy human subjects (*32*) and patients with lung disease (*33*), and is being evaluated in a European phase 2 clinical trial for COVID-19 managed by Apeiron Biologics. Peptide derivatives of ACE2 are also being explored as cell entry inhibitors (*34*).

Because human ACE2 has not evolved to recognize SARS-CoV-2 S, we hypothesized that mutations may be found that increase affinity. The coding sequence of full-length ACE2 with an N-terminal c-MYC epitope tag was diversified to create a library containing all possible single–amino acid substitutions at 117 sites that span the interface with S and the angiotensin peptide-binding cavity. S binding is independent of ACE2 catalytic activity (*35*) and occurs on the outer surface of ACE2 (*17*, *18*), whereas angiotensin substrates bind within a deep cleft that houses the active site (*36*).

The ACE2 library was transiently expressed in human Expi293F cells under conditions that typically yield no more than one coding variant per cell, providing a tight link between genotype and phenotype (*37*, *38*). Cells were then incubated with a subsaturating dilution of medium containing the RBD of SARS-CoV-2 fused to superfolder green fluorescent protein [sfGFP; (*39*)] (fig. S1A). Dual-color flow cytometry measurements show that amounts of bound RBD-sfGFP correlate with surface expression levels of MYC-tagged ACE2. Compared with cells expressing wild-type ACE2 (fig. S1C), many variants in the ACE2 library fail to bind RBD, whereas a smaller number of ACE2 variants showed higher binding signals (fig. S1D). Populations of cells that express ACE2 variants at the cell surface with high (“nCoV-S-High”) or low (“nCoV-S-Low”) binding to RBD were collected by fluorescence-activated cell sorting (FACS) (fig. S1D). During FACS, the fluorescence signal for bound RBD-sfGFP continuously declined, requiring the collection gates to be regularly updated to “chase” the relevant populations. This is consistent with RBD dissociating during the experiment.

**Fig. 1:**
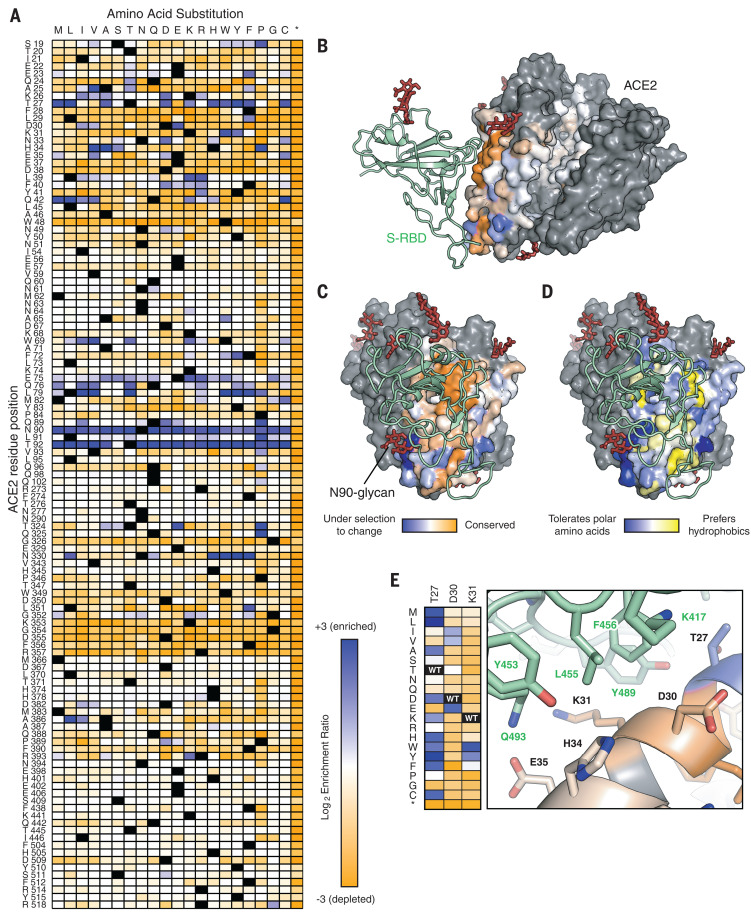
Sequence preferences of ACE2 residues for high binding to the RBD of SARS-CoV-2 S. (**A**) Log_2_ enrichment ratios from the nCoV-S-High sorts are plotted from depleted or deleterious (orange) to enriched (dark blue). ACE2 primary structure is shown on the vertical axis, amino acid substitutions are indicated on the horizontal axis. Wild-type amino acids are in black. Asterisk (*) denotes stop codon. (**B**) Conservation scores are mapped to the structure (Protein Data Bank 6M17) of RBD (green ribbon)–bound protease domain (surface), oriented with the substrate-binding cavity facing the reader. Residues conserved for RBD binding are shown in orange; mutationally tolerant residues are in pale colors; residues that are hot spots for enriched mutations are in blue; and residues maintained as wild type in the ACE2 library are in gray. Glycans are depicted as dark red sticks. (**C**) Viewed looking down on to the RBD interaction surface. (**D**) Average hydrophobicity-weighted enrichment ratios are mapped to the structure, with residues tolerant of polar substitutions in blue and residues that prefer hydrophobics in yellow. (**E**) A magnified view of the ACE2–RBD interface [colored as in (B) and (C)]. Heat-map plots log_2_ enrichment ratios from the nCoV-S-High sort. Abbreviations for the amino acid residues are as follows: A, Ala; C, Cys; D, Asp; E, Glu; F, Phe; G, Gly; H, His; I, Ile; K, Lys; L, Leu; M, Met; N, Asn; P, Pro; Q, Gln; R, Arg; S, Ser; T, Thr; V, Val; W, Trp; and Y, Tyr.

In an approach known as deep mutagenesis (*40*), the enrichment or depletion of all 2340 coding mutations in the library was determined by comparing the frequencies of transcripts in the sorted populations to sequence frequencies in the naïve plasmid library (Fig. 1A). Enrichment ratios and residue conservation scores closely agree between two independent FACS experiments (fig. S2). Enrichment ratios and conservation scores in the nCoV-S-High sorted cells tend to be negatively correlated with the nCoV-S-Low sorted cells, with the exception of nonsense mutations that do not express and were therefore depleted from both populations (fig. S2). Most, but not all, nonsynonymous mutations in ACE2 did not eliminate surface expression (fig. S2). The library is biased toward solvent-exposed residues and has few substitutions of buried hydrophobic residues that might have greater effects on plasma membrane trafficking (*38*).

Mapping the experimental conservation scores from the nCoV-S-High sorted cells to the structure of RBD-bound ACE2 (*17*) shows that residues buried in the interface tend to be conserved, whereas residues at the interface periphery or in the substrate-binding cleft are mutationally tolerant (Fig. 1, B and C). The region of ACE2 surrounding the C-terminal end of the ACE2 α1 helix and β3-β4 strands has a weak tolerance for polar residues, whereas amino acids at the N-terminal end of α1 and the C-terminal end of α2 are preferentially hydrophobic (Fig. 1D), likely in part to preserve hydrophobic packing between α1-α2. These discrete patches contact the globular RBD fold and a long protruding loop of the RBD, respectively.

Two ACE2 residues, N90 and T92 that together form a consensus N-glycosylation motif, are notable hot spots for enriched mutations (blue in Fig. 1A). Indeed, all substitutions of N90 and T92, with the exception of T92S, which maintains the N-glycan, are highly favorable for RBD binding, and the N90-glycan is thus predicted to partially hinder the S–ACE2 interaction. These results may depend on the chemical nature of glycan moieties attached in different cell types.

Mining the data identifies many ACE2 mutations that are enriched for RBD binding. It has been proposed that natural ACE2 polymorphisms are relevant to COVID-19 pathogenesis and transmission (*41*, *42*), and the mutational landscape provided here will facilitate analyses to test this. At least a dozen ACE2 mutations at the interface enhance RBD binding, and the molecular basis for affinity enhancement can be rationalized from the RBD-bound ACE2 cryo–electron microscopy (EM) structure (Fig. 1E) (*17*): Hydrophobic substitutions of ACE2-T27 increase hydrophobic packing with aromatic residues of S, ACE2-D30E extends an acidic side chain to reach S-K417, and aromatic substitutions of ACE2-K31 contribute to an interfacial cluster of aromatics. A search for affinity-enhancing mutations in ACE2 using targeted mutagenesis recently identified D30E (*43*), providing independent confirmation of this result.

There are also enriched mutations in the second shell and farther from the interface that do not directly contact S but instead have putative structural roles. For example, proline substitutions were enriched at five library positions (S19, L91, T92, T324, and Q325) where they might entropically stabilize the first turns of helices. Proline was also enriched at H34 where it may enforce the central bulge in α1, and multiple mutations were enriched at buried positions where they will change local packing (e.g., A25V, L29F, W69V, F72Y, and L351F). The selection of ACE2 variants for high binding signal therefore reports not only on affinity, but also on presentation at the membrane of folded structure recognized by SARS-CoV-2 S. Whether these mutations selectively stabilize a virus-recognized local structure in ACE2 versus the global protein fold is unclear.

Thirty single–amino acid substitutions highly enriched in the nCoV-S-High sorted cells were validated by targeted mutagenesis (fig. S3). Binding of RBD-sfGFP to full-length ACE2 mutants measured by dual-color flow cytometry (fig. S3) increased compared with that of the wild type, yet improvements were small and most apparent on cells expressing low amounts of ACE2. Differences in ACE2 expression between the mutants also correlated with total amounts of bound RBD-sfGFP (fig. S3C), demonstrating the need for caution in interpreting deep mutational scan data as mutations may affect both activity and expression. To rapidly assess mutations in a soluble format, we fused the ACE2 protease domain to sfGFP. Expression levels of sACE2-sfGFP were evaluated qualitatively by fluorescence (fig. S4A), and binding to full-length S expressed at the plasma membrane was measured by flow cytometry (fig. S4B). A single substitution (T92Q) in the N90 glycosylation motif gave a modest increase in binding signal, which was confirmed by analysis of purified protein (fig. S5). Focusing on the most highly enriched substitutions in the nCoV-S-High sorted cells that were also spatially segregated to minimize negative epistasis (*44*), combinations of mutations were expressed, and these gave sACE2 large increases in S binding (materials and methods, table S1, and fig. S4B). Unexplored combinations of mutations may have even greater effects.

A single variant, sACE2.v2, was chosen for purification and further characterization (fig. S6). This variant was selected because it was well expressed as a sfGFP fusion and it maintains the N90-glycan, thus presenting a surface that more closely matches that of native sACE2 to minimize immunogenicity. The yield of sACE2.v2 was lower than that of the wild-type protein, and by analytical size exclusion chromatography (SEC), a small fraction of sACE2.v2 was found to aggregate after incubation at 37°C (fig. S6D). Otherwise, sACE2.v2 was indistinguishable from the wild type by SEC (fig. S6C).

**Fig. 2:**
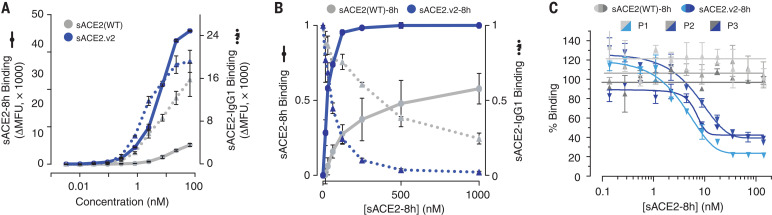
A variant of sACE2 with high affinity for S. (**A**) Expi293F cells expressing S were incubated with purified wild-type sACE2 (gray) or sACE2.v2 (blue) fused to 8His (solid lines) or IgG1-Fc (broken lines). Bound protein was detected by flow cytometry. Data are mean fluorescence units (MFU) of the total cell population after subtraction of background autofluorescence. *n* = 2 replicates, error bars represent range. (**B**) Binding of 100 nM wild-type sACE2-IgG1 (broken lines) was competed with wild type sACE2-8h (solid gray line) or sACE2.v2-8h (solid blue line). The competing proteins were added simultaneously to cells expressing S, and relative bound protein was detected by flow cytometry. *n* = 2 replicates, error bars represent range. (**C**) Competition for binding to immobilized RBD in an ELISA between serum IgG from COVID-19 patients versus wild-type sACE2-8h (gray) or sACE2.v2-8h (blue). Three different patient sera were tested (P1 to P3 in light to dark shades). Data are mean ± SEM, *n* = 2 replicates.

In flow cytometry experiments using the purified 8His-tagged protease domain, sACE2.v2-8h, but not wild type, was found to bind strongly to full-length S at the cell surface, suggesting that wild-type sACE2 has a faster off-rate that causes dissociation during sample washing (Fig. 2A and fig. S7). Differences between wild type and the variant were less pronounced in the context of an immunoglobulin G1 (IgG1)–Fc fusion (Fig. 2A and fig. S7), indicating that avidity masks gains in binding of the mutant, again consistent with off-rate differences between wild type and variant sACE2. Soluble ACE2.v2-8h outcompetes wild-type sACE2-IgG1 for binding to S-expressing cells, yet wild-type sACE2-8h does not outcompete sACE2-IgG1, even at 10-fold higher concentrations (Fig. 2B). Furthermore, only engineered sACE2.v2-8h effectively competed with anti-RBD IgG in serum from three COVID-19–positive patients when tested by enzyme-linked immunosorbent assay (ELISA) (Fig. 2C). The observation that up to 80% inhibition was achieved at saturation with sACE2.v2-8h indicates that most antibodies against RBD were directed at the receptor-binding site. Finally, biolayer interferometry (BLI) showed that sACE2.v2 has 65-fold higher affinity than the wild-type protein for immobilized RBD, almost entirely due to a slower off-rate (table S2 and fig. S6, E and F).

To address the decreased expression of sACE2.v2, it was hypothesized that the mutational load is too high. In second-generation designs, each of the four mutations in sACE2.v2 was reverted back to the wild-type identity (table S1), and binding to full-length S at the cell surface remained high (fig. S8A). One of the variants (sACE2.v2.4 with mutations T27Y, L79T and N330Y) was purified with even higher yields than that of the wild type and displayed tight nanomolar binding to the RBD (fig. S8).

**Fig. 3:**
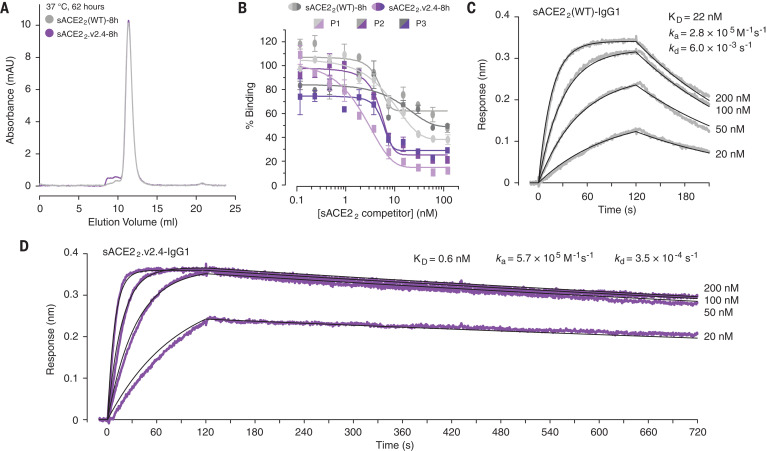
A dimeric sACE2 variant with improved properties for binding viral spike. (**A**) Analytical SEC of wild-type sACE2_2_-8h (gray) and sACE2_2_.v2.4-8h (purple) after incubation at 37°C for 62 hours. (**B**) ELISA analysis of serum IgG from COVID-19 patients (P1 to P3 in light to dark shades) binding to RBD. Dimeric sACE2_2_(WT)-8h (gray) or sACE2_2_.v2.4-8h (purple) are added to compete with antibodies recognizing the receptor-binding site. Concentrations are based on monomeric subunits. Data are mean ± SEM, *n* = 2 replicates. (**C**) RBD-8h association (*t* = 0 to 120 s) and dissociation (*t* > 120 s) with immobilized sACE2_2_(WT)-IgG1 measured by BLI. (**D**) BLI kinetics of RBD-8h binding to immobilized sACE2_2_.v2.4-IgG1.

The ACE2 construct was lengthened to include the neck or dimerization domain, yielding a stable dimer (Fig. 3A) referred to here as sACE2_2_, which binds with high avidity to S on the cell surface or immobilized RBD on a biosensor (fig. S9). Compared to the wild type, dimeric sACE2_2_.v2.4 competes more effectively with IgG present in serum from COVID-19 patients (Fig. 3B). The engineered dimer may be useful in assessing serum or plasma (e.g., for convalescent plasma therapies) for concentrations of the most effective SARS-CoV-2 neutralizing antibodies (*45*). By immobilizing sACE2_2_-IgG1 (fig. S10) to a biosensor surface and incubating it with monomeric RBD-8h as the analyte, we determined the dissociation constant *K*_D_ of RBD for wild-type sACE2_2_ to be 22 nM (Fig. 3C), in close agreement with previous reports (*8*, *46*), whereas sACE2_2_.v2.4 bound with 600 pM affinity (Fig. 3D). This compares favorably with results from recently isolated monoclonal antibodies (*22*–*28*).

**Fig. 4:**
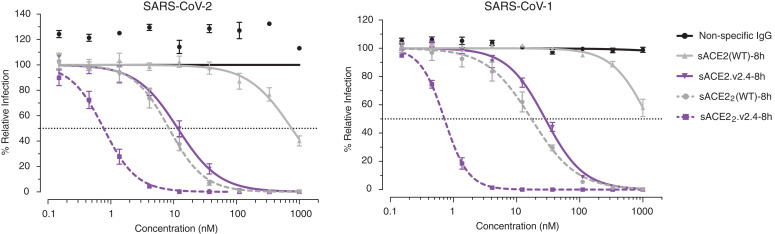
Enhanced neutralization of SARS-CoV-1 and -2 by engineered receptors. In a microneutralization assay, monomeric (solid lines) or dimeric (broken lines) sACE2(WT)-8h (gray) or sACE2.v2.4-8h (purple) were preincubated with virus before adding to VeroE6 cells. Concentrations are based on monomeric subunits. Data are mean ± SEM of *n* = 4 replicates.

The efficacy of monomeric sACE2.v2.4 in neutralizing SARS-CoV-2 infection of cultured VeroE6 cells exceeded that of the wild-type protein by nearly two orders of magnitude (Fig. 4), consistent with the biochemical binding data. Wild-type, dimeric sACE2_2_ is itself two orders of magnitude more potent than the monomeric subunit, indicating strong, avid interactions with spike on the virion surface, and dimeric sACE2_2_.v2.4 is yet again more potent with a subnanomolar median inhibitory concentration (Fig. 4). Dimeric sACE2_2_.v2.4 also potently neutralizes SARS-CoV-1, despite no consideration of SARS-CoV-1 S structure or sequence during the engineering process, and it is possible that the decoy receptor will neutralize diverse ACE2-utilizing coronaviruses that have yet to cross over to humans.

To improve safety, we manufactured untagged sACE2_2_.v2.4 in ExpiCHO-S cells (fig S11A) and found it to be stable after incubation at 37°C for 6 days (fig S11B). The protein competes with wild-type sACE2_2_-IgG1 for cell-expressed S (fig. S11C) and binds with tight avidity to immobilized RBD (fig S11D). In addition to inhibiting virus entry, recombinant sACE2 may have a second therapeutic mechanism: proteolysis of angiotensin II (a vasoconstrictive peptide hormone) to relieve symptoms of respiratory distress (*30*, *31*). Soluble ACE2_2_.v2.4 is found to be catalytically active, albeit with reduced activity (fig. S12). Whether this confers any therapeutic advantage or disadvantage over wild-type sACE2 remains to be seen.

With astonishing speed, the scientific community has identified multiple candidates for the treatment of COVID-19, especially monoclonal antibodies with exceptional affinity for protein S. Our work shows how comparable affinity can be engineered into the natural receptor for the virus, while also providing insights into the molecular basis for initial virus-host interactions.

## Supplementary Materials

science.sciencemag.org/content/369/6508/1261/suppl/DC1 Materials and Methods Figs. S1 to S12 Tables S1 and S2 References (*47*–*49*) MDAR Reproducibility Checklist Data File S1

## Appendix

View/request a protocol for this paper from *Bio-protocol*.
